# Long-Range Correlations in Stride Intervals May Emerge from Non-Chaotic Walking Dynamics

**DOI:** 10.1371/journal.pone.0073239

**Published:** 2013-09-23

**Authors:** Jooeun Ahn, Neville Hogan

**Affiliations:** 1 Department of Mechanical Engineering, Massachusetts Institute of Technology, Cambridge, Massachusetts, United States of America; 2 Department of Brain and Cognitive Sciences, Massachusetts Institute of Technology, Cambridge, Massachusetts, United States of America; University of California, Merced, United States of America

## Abstract

Stride intervals of normal human walking exhibit long-range temporal correlations. Similar to the fractal-like behaviors observed in brain and heart activity, long-range correlations in walking have commonly been interpreted to result from chaotic dynamics and be a signature of health. Several mathematical models have reproduced this behavior by assuming a dominant role of neural central pattern generators (CPGs) and/or nonlinear biomechanics to evoke chaos. In this study, we show that a simple walking model without a CPG or biomechanics capable of chaos can reproduce long-range correlations. Stride intervals of the model revealed long-range correlations observed in human walking when the model had moderate orbital stability, which enabled the current stride to affect a future stride even after many steps. This provides a clear counterexample to the common hypothesis that a CPG and/or chaotic dynamics is required to explain the long-range correlations in healthy human walking. Instead, our results suggest that the long-range correlation may result from a combination of noise that is ubiquitous in biological systems and orbital stability that is essential in general rhythmic movements.

## Introduction

Though human walking is highly stereotyped, the stride intervals fluctuate from one stride to the next with a measurable variance. Interestingly, in healthy adult walking, the variations in stride intervals exhibit long-range correlations [1], [2]. This observation has supported the hypothesis that the step-to-step variation exhibits fractal-like behavior rather than uncorrelated stochastic noise simply superimposed on regular dynamics. The importance of this long-range correlation has been further emphasized since several studies reported that age and neurological disorders decrease the correlations [3], [4], [5], suggesting that long-range correlations may indicate a healthy locomotor system. Interesting similarities are found in other rhythmic activities of normal neural and cardiac systems; prior studies reported fractal-like behaviors in normal heart beating and brain activity that alter due to diseases such as heart attack or epileptic seizure [6], [7], [8], [9].

Because of the deep connection between fractals and chaos, the observed fractal-like behaviors have commonly been interpreted as evidence of chaos in healthy biological systems. Pool proposed that “chaos may provide a healthy flexibility of the heart, brain, and other parts of the body” [10]. Goldberger et al. mentioned that many pathologies exhibit increasingly periodic behaviors and loss of the chaotic variability that is observed in healthy biological systems [6]. The proposal that chaos is “healthy” has motivated studies to develop clinical measures of health based on methods of nonlinear dynamics and time series analysis [7], [8], [9]. In the study reported here, we question this appealing proposal, at least in the context of normal human walking. We show that nonlinear dynamics capable of chaos may not be necessary to explain the phenomenon of long-range correlation. The ubiquitous neuro-muscular noise combined with essential but non-chaotic biomechanics may be sufficient to explain the observed long-range correlations in stride intervals.

In an attempt to address the origin of the potentially important but counter-intuitive long-range correlations in stride intervals, several mathematical models have been proposed [1], [11], [12], [13], [14]. The relation among long-range correlation, fractal-like behaviors and chaos motivated most of the previous models to include nonlinear oscillators such as neural central pattern generators (CPGs) that can result in fractal-like behaviors. Hausdorff et al. successfully reproduced the observed dynamics of stride intervals by introducing “memory” into a CPG; when only certain transitions from mode to mode were allowed inside a CPG, the resulting stride intervals had long-range correlations [1]. An extension of this model could encapsulate the difference in gait dynamics between children and adults [11]. West et al. further reproduced the pronounced long-range correlations of slow and fast walking and the loss of long-range correlations in metronomic walking by introducing a “super CPG model”; they assumed that impulses from correlated firing of neural centers regulated the intrinsic frequency of a forced van der Pol oscillator whose actual period coincided with the stride interval [12].

Unlike most of the models that attribute long-range correlations to specific neural oscillator mechanisms, a model by Gates et al. showed that long-range correlations in stride intervals may emerge from biomechanics [14]. They added noisy neural input to a simplified passive dynamic walker presented by Garcia et al [15], and argued that the long-range correlations may result from a combination of noisy neural signals and highly nonlinear biomechanics. However, the original model by Garcia et al. is already able to produce chaotic and fractal behaviors which may result in long-range correlations [15]. It is therefore still unclear whether nonlinear biomechanics capable of chaotic dynamics is essential to generate long-range correlations.

Here, we present a highly simplified walking model that can reproduce the long-range correlations observed in stride intervals without complex peripheral dynamics. The model is deliberately formulated to be incapable of chaotic dynamics and does not include a self-sustaining neural oscillator such as a CPG. Nevertheless, with uncorrelated stochastic actuation noise, this simple one degree of freedom walking model can reproduce long-range correlations in stride intervals. A physical interpretation of long-range correlation is that the current stride can affect a future stride even after many strides. In a limiting case where the effect of the current stride is never forgotten, the stride intervals may approach Brownian noise. Our premise is that this memory effect may be related to orbital stability, which determines how fast a perturbation can be forgotten. Based on this premise, we propose a hypothesis that moderate orbital stability may permit long-range correlations such as have been observed in human walking.

## Model

### General Description

**Figure 1:**
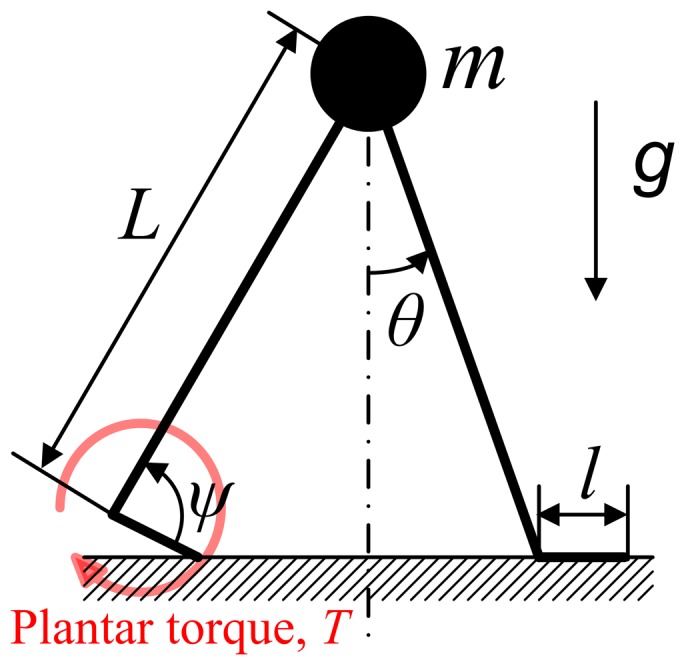
A schematic of the walking model. A point mass is restrained by rigid massless legs. The trailing ankle is actuated as a pre-loaded spring released at the beginning of double stance. The hip joint and the leading ankle do not exert any torque.

The model presented here is an updated version of the model presented in [16]. A schematic of the model defining its variables and parameters is shown in Fig. 1. A point mass moves in a vertical plane under the influence of gravity, restrained by rigid massless legs. The swing leg can be moved instantaneously in front of the mass. Scuffing (contact of the swing leg with the ground) is ignored. Each leg has two joints—a hip and an ankle. Ankle actuation provides propulsion whereas the hip joint is assumed to be a frictionless pivot, which cannot apply any torque. However, we assume that the angle between the legs is always reset as 2*θ*_0_ at the beginning of a step. Due to the assumption of massless legs, resetting the angle between the legs does not consume any energy.

**Figure 2:**
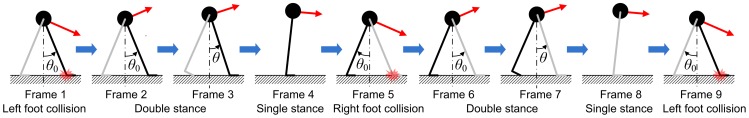
One stride of the walking model. The end and beginning of a step is the moment when the leading foot collides with ground (Frame 1, 5 and 9). During double stance the model moves as four linked bars (Frame 2, 3, 6 and 7). During single stance the model moves as an inverted pendulum (Frame 4 and 8).

Sequential configurations of the model during one stride cycle are depicted in Fig. 2. At the collision of the leading foot with the ground (Frame 1), the velocity of the point mass changes instantaneously; the direction changes by 2*θ*_0_ and the magnitude is reduced by cos2*θ*_0_ by the angular momentum principle (Frame 2). Immediately after the collision, the model is in double stance and the trailing ankle is actuated. During double stance the model behaves as an actuated four-bar linkage (Frame 2 and 3). The ankle of the leading leg acts as a hinged joint during double stance and the following single stance phase (Frame 4). We assume that the ankle torque during double stance is determined by a linear torsional spring as
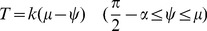
(1)where *T* is plantar ankle torque at the trailing ankle, *ψ* is ankle angle that is positive towards plantar flexion, and *μ* is maximal plantar flexion angle.

The modification in this study over that presented in [16] is to add stochastic variability to the ankle torque constant, *k*. At every step, *k* is updated as a random variable whose probability density function has a normal distribution with mean of *k*_0_ and variance of *σ* ^2^. The variance *σ* ^2^ represents the noise level added to the system. The torque becomes zero when *ψ* reaches *μ*. By virtue of the zero mass of the feet, the trailing foot pushes on the ground only as long as the actuation torque is positive; double stance ends at the moment when the ankle torque becomes zero, or equivalently when *ψ* reaches *μ*. During the following single stance (Frame 4 and 8 in Fig. 2), there is no actuation torque, and the dynamics of the swing leg is irrelevant because it has no mass; the model acts like an inverted pendulum hinged at the ankle of the stance leg. A step cycle ends when the hip angle *θ* reaches -*θ*_0_, its value at the foot-ground collision, and the next step follows (Frame 1, 5, and 9); a stride consists of two consecutive steps.

For the deterministic version of the model, the equations of motion and the ground reaction forces are derived, and the existence and stability of a periodic gait are analyzed in [16]. Here, we briefly recapitulate the return map analysis in [16] to emphasize the existence, uniqueness and asymptotic stability of a limit cycle of the model. We used the concept of a step-to-step function whose input and output are state variables at the beginning of one step and at the beginning of the next step respectively [17]. Mathematically this step-to-step function is a return map. Using the work-energy principle, the step-to-step function of the model is expressed as
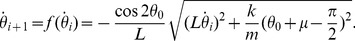
(2)Where 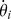 is the angular velocity of the leading leg right after the *i* ^th^ foot-ground collision. This return map has a unique fixed point that satisfies 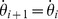, and any initial condition converges monotonically to this fixed point as the number of steps increases. (Proof in Appendix S1.) Therefore, neither bifurcation (period doubling) nor chaotic behavior is possible. The model has a unique stable limit cycle and the stability is determined solely by the parameter *θ*_0_. The orbital stability of a limit cycle can be quantified by Floquet multipliers [18], [19], [20]. In general, Floquet multipliers are complex valued eigenvalues of a linearized return map at its fixed point. If all the Floquet multipliers are inside a unit circle on the complex plane, the limit cycle is asymptotically stable. If there exists a Floquet multiplier outside the unit circle, the limit cycle is unstable. This specific model has only one Floquet multiplier which is cos^2^2*θ*_0_. Therefore, the limit cycle is always asymptotically stable except in the limit where *θ*_0_ approaches zero.

### Parameter Values

Parameter values are summarized in Table 1. Mass, leg length, foot length and maximal plantar extension angle were chosen to approximate morphological data of human adults. Two values of *θ*_0_ were selected as *π*/12 and *π*/6 radian to investigate how the model behavior changed when its orbital stability changed. For each *θ*_0_, the nominal ankle actuation constant *k*_0_ was chosen to match the average speed of the model with that of normal human walking, which is 1.35 m/s on average [21]. For each *θ*_0_, the variance of ankle actuation *σ* ^2^ was chosen to match the model's coefficient of variance (COV) of walking cadence with that of normal human walking, which is 3% [1].

**Table 1:** Parameter values for the model.

Parameter	Meaning	Value
*m*	mass	80 kg
*L*	leg length	1 m
*l*	foot length	0.2 m
*g*	gravitational acceleration	9.81 m/s^2^
*μ*	maximal plantar extension of the ankle	2.576 rad

## Methods

To compare results with the original study by Hausdorff et al. [1], we faithfully followed the analysis method they used. Two indices that distinguish between white noise, Brownian noise and time series with long-range correlations were obtained from detrended fluctuation analysis (DFA) and power spectral analysis. Numerical simulation was implemented in Matlab using the Simulink toolbox (Mathworks Inc.). Numerical integration by the Runge-Kutta method was performed with a fixed step size of 10^−4^. The validity of the numerical simulation was checked by repeating simulations with a tenfold smaller step size. Every statistical analysis was performed at a significance level of 5%.

### Detrended Fluctuation Analysis (DFA)

DFA is a method to determine long-range correlations which was introduced by Peng et al. [22]. A total number N of stride intervals are integrated to generate a time series y as
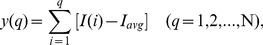
(3)where I(i) is the i^th^ stride interval and I_avg_ is the average of the stride intervals. The time series y is divided into windows of length n samples. For each window size n, a local least squares line fit is calculated in every window. Let the fitted value be y_n_(q). The average fluctuation of y with respect to the locally best-fit line is calculated for each window size n as
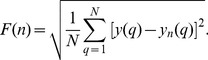
(4)The slope of the least square fit line relating log F(n) and log n is the scaling exponent α, which becomes 0.5 for white noise, 1.5 for Brownian noise, and a value between 0.5 and 1 for time series with long-range correlations. To compare with the original experimental observation in [1], the model walked 500 strides. To further investigate the fluctuation structure of stride intervals for longer simulation, the model also walked 3,000 strides, which approximate the number of strides in one hour of walking. We additionally simulated 100,000 strides to examine the structure of the stride interval time series for even longer walking. In each case, the window size n varied from 4 to half of the maximum stride number so that log n had increments of 0.1. For 100,000 stride walking, window sizes larger than 1,000 strides (1,000≤n≤50,000) were additionally considered to inspect any change due to the significant increase of stride number. We ran 20 simulations of 500, 3,000, and 100,000 stride walking, and the scaling exponent α was evaluated for each simulation.

### Power Spectral Analysis

Another index of long-range correlations was calculated from the power spectrum of the time series. Following the method presented in [1], we obtained the power spectrum of the time series, S(f) as the square of the amplitudes of the Fourier spectrum, where f is the inverse of stride number. The slope of a regression line relating log S(f) and log f was evaluated. This slope becomes 0 for white noise, −1 for 1/f noise and −2 for Brownian noise. It is more common to use β (−1×the slope) as a metric that characterizes the power spectrum. The index β was evaluated for each of 20 simulations (500, 3,000, or 100,000 strides per simulation). For simulations of 500 strides, f varied from 0.01 to 0.3 (stride number)^−1^ following the method in [1]. For simulations of 3,000 strides, f varied from 1/600 to 0.3 (stride number)^−1^ to account for the factor of 6 increase of stride number. For simulations of 100,000 strides, f varied from 2×10^−5^ to 0.3 (stride number)^−1^, and the interval from 2×10^−5^ to 0.01 (stride number)^−1^ was additionally considered to investigate any change due to the increase of stride number.

### Extreme Cases and Shuffled Strides for Comparison

**Figure 3:**
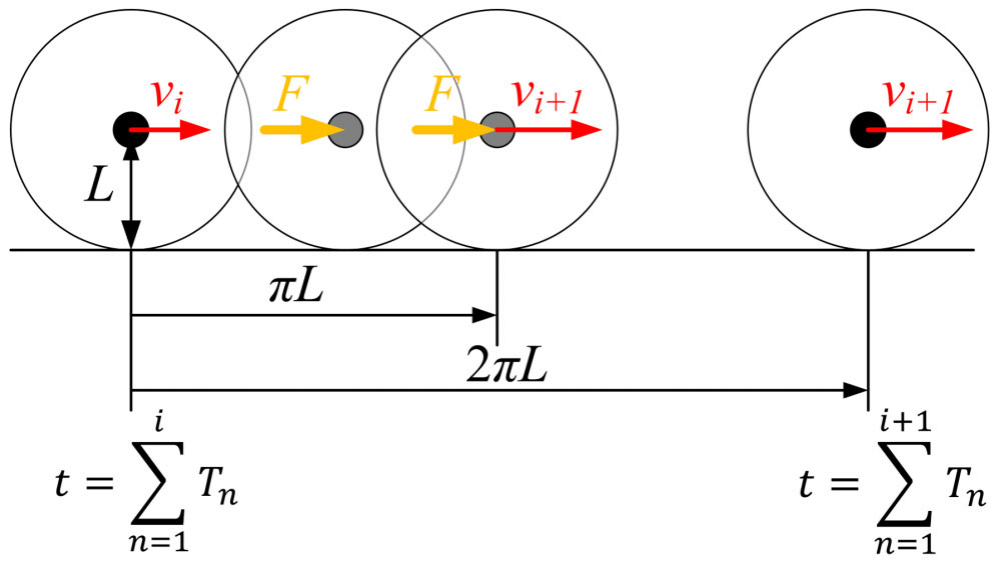
One stride of the model in the limit of zero *θ*_0_. *L*, *F*, *v_i_* and *T_i_* are the radius of the disk, the stochastic force with zero mean applied to the center of mass while the disk is rolling from 0 to *π* radian, the velocity of the disk at the end of *i* ^th^ cycle, duration of *i* ^th^ cycle, respectively. The velocity *v_i_*_+1_ is determined right after the disk rolls by *π* radian because the stochastic force is no longer applied to the disk hence momentum is conserved.

For comparison, the two indices α and β were investigated for two extreme cases. First, we artificially generated a time series of stride intervals as a normally distributed random process with mean of 1.23 s and standard deviation of 0.0369 s. The mean and the standard deviation were chosen to match the experimental data in [1]. The random stride intervals were generated 500 times and 3,000 times in each of 20 simulations. Second, we considered the model behavior in the limit as θ_0_ approaches zero. Physically, a rimless spoked wheel model approaches a rolling disk as the number of spokes approaches infinity and the angle between the neighboring spokes approaches zero [23]. In the limit of infinitesimal θ_0_, our model likewise approaches a rolling disk with mass m and zero moment of inertia. In this limit, to yield non-diverging gait, the ankle actuation has to converge to zero. For this case of infinitesimal θ_0_, we re-defined a stride interval as a time interval required for the disk to recover its orientation by rotating 2π radian. The initial velocity of the rolling disk was chosen to match the nominal period with 1.23 s, the average stride interval of human walking. The variability of ankle torque in the original model was replaced with a normally distributed stochastic force applied to the point mass. Considering that ankle actuation provides propulsion only during double stance phase in the model, and double stance occupies approximately 50% of the gait cycle in human walking, the stochastic force vanished when the disk rolled by π radian. The normally distributed stochastic force had zero mean. In this limiting case, the effect of the current stride is never forgotten. Therefore the stride intervals approach Brownian noise whose variance increases as stride number increases. We chose the variance of the stochastic force to be small enough so that the COV of the re-defined stride intervals over 500 cycles matched that of normal human walking, which is 3% [1]. This model of the limit of zero θ_0_ is depicted in Fig. 3. The indices α and β were evaluated in 20 simulations each of which had 500 or 3,000 cycles.

To confirm that any difference in the scaling exponents (α and β) resulted from the temporal structure rather than a specific distribution of the noise, we applied the method of surrogate data introduced by Theiler et al. [24]. Both α and β of shuffled time series of each realization were evaluated and statistically compared with those of original data.

## Results

**Figure 4:**
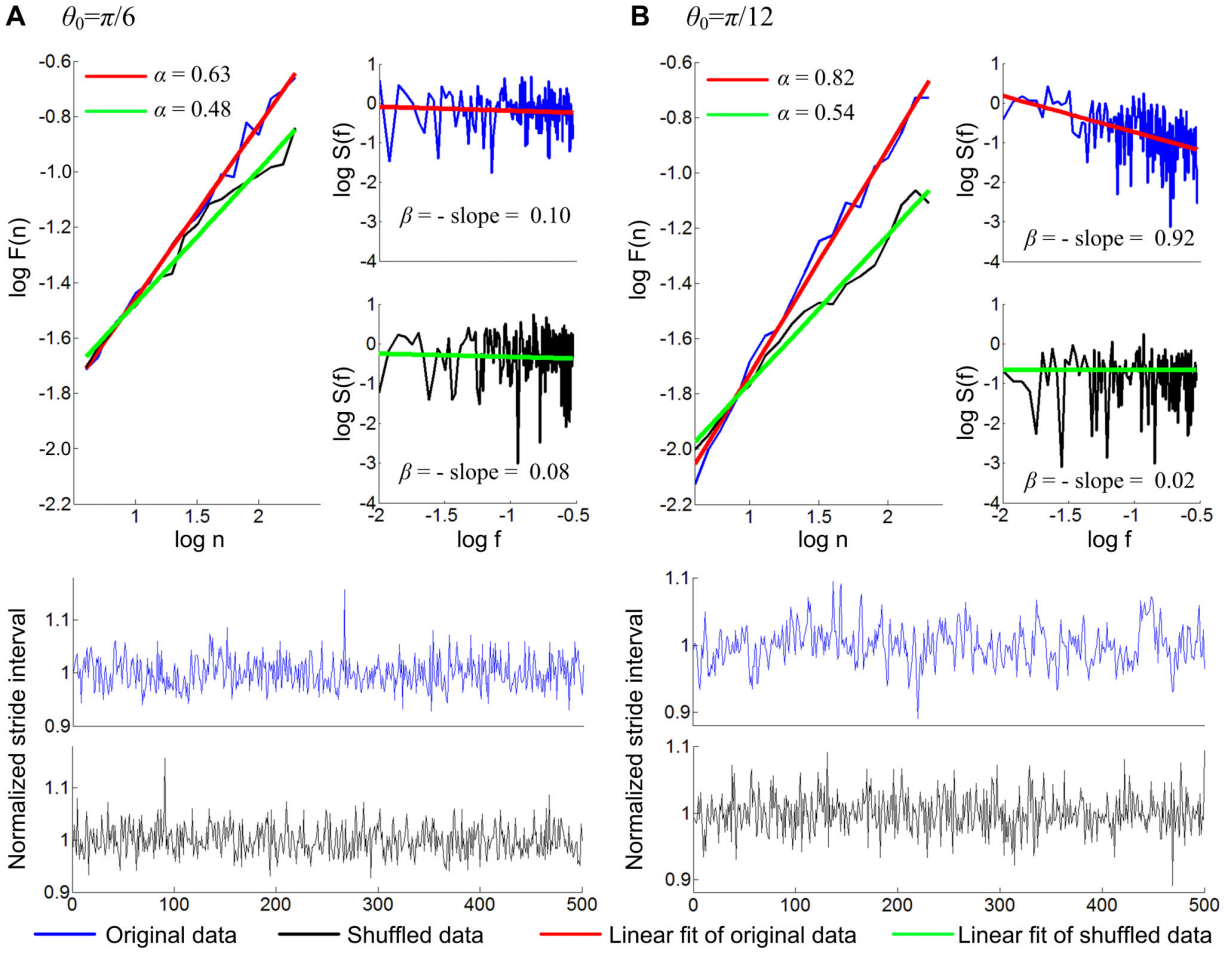
Scaling exponents *α* and *β* for 500 strides of walking with two different values of *θ*_0_. The leading leg angle *θ*_0_ defines the Floquet multiplier as cos^2^2*θ*_0_, and determines how fast the perturbed dynamics converges to the nominal limit cycle. Small *θ*_0_ makes the Floquet multiplier close to unity yielding slow convergence, and large *θ*_0_ makes the Floquet multiplier close to zero yielding fast convergence. Both *α* and *β* are slightly different from those of the shuffled data with no correlation when *θ*_0_ is *π*/6 and the model yields relatively strong orbital stability (Floquet multiplier = 0.25). The difference in the scaling exponents between the original time series and their shuffled counterparts becomes much more prominent when *θ*_0_ is *π*/12 and the attraction to the limit cycle becomes relatively weak (Floquet multiplier = 0.75); *α* and *β* become similar to those of human walking with long-range correlations with clear difference from those of the shuffled data. The bottom panels show time series of the normalized stride intervals (stride intervals divided by their mean value). The structure of the time series changed due to shuffling particularly when *θ*_0_ is *π*/12.

**Figure 5:**
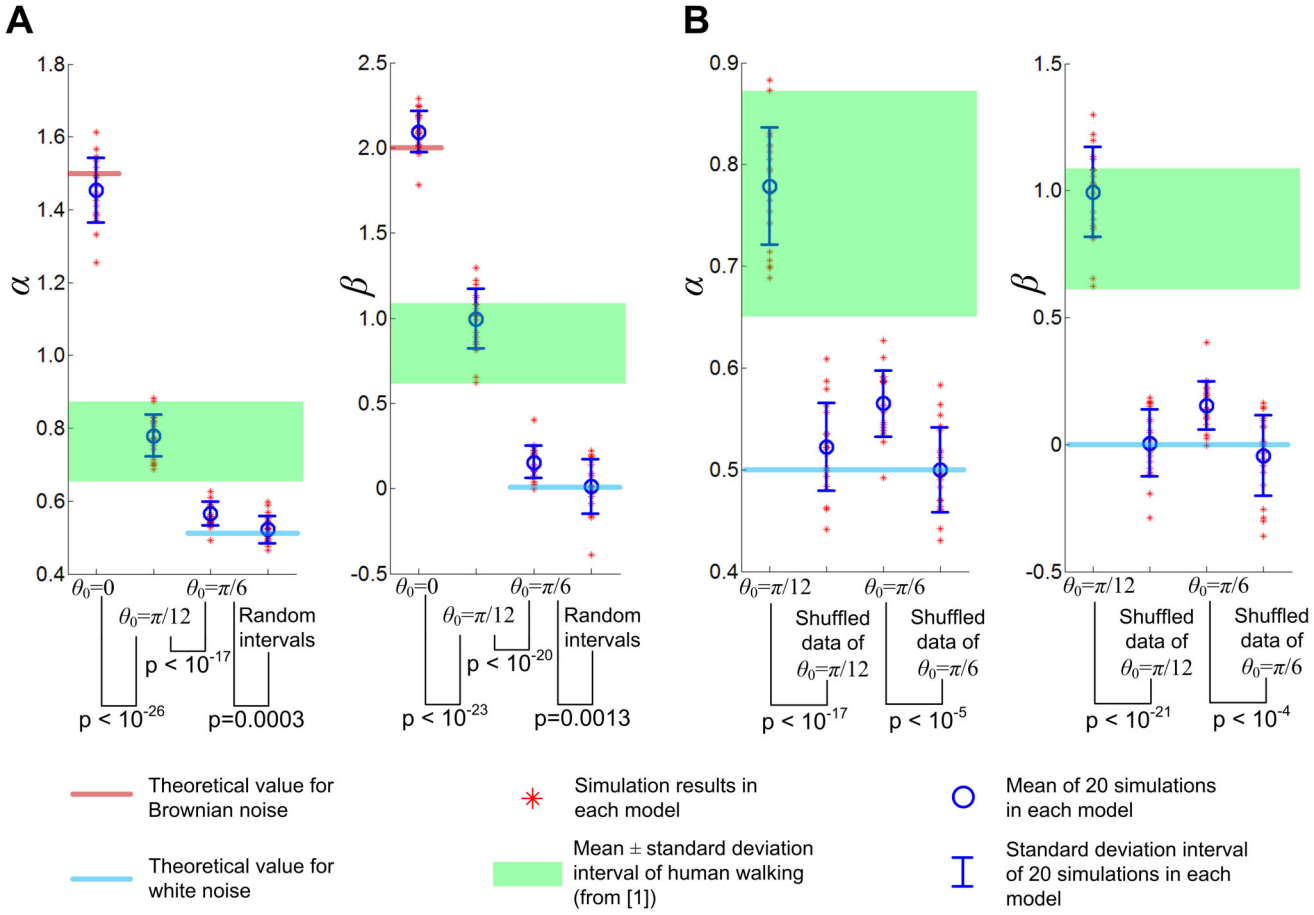
Distribution of scaling exponents *α* and *β* when the model walked 500 strides. In A, the scaling exponents *α* and *β* were evaluated for each of 20 simulations in four different cases. In addition to cases with two different values of *θ*_0_ (*π*/12 and *π*/6), two extreme cases were investigated for comparison. When the stride intervals are artificially generated as a random variable from a normal distribution, they become white noise. The stride intervals with *θ*_0_ = *π*/6 show a slight indication of long-range correlations. When *θ*_0_ = *π*/12, a similar leg angle to that of normal human walking, the stride intervals present evident long-range correlations with similar *α* and *β* to those observed in human walking. When *θ*_0_ approaches zero and the model becomes a rolling disk, the stride intervals approach Brownian noise. In B, the distributions of *α* and *β* for *θ*_0_ = *π*/12 and *θ*_0_ = *π*/6 are compared with those of their shuffled counterparts. The scaling exponents of the original time series are significantly different from those of the shuffled data which approximate *α* and *β* of white noise. This confirms that temporal structure, rather than a specific distribution of variability, gives rise to the long-range correlation in stride intervals of the walking model.

Hausdorff et al. reported that *α* = 0.76±0.11 (SD) for 10 subjects, and *β* = 0.83±0.23 for 8 subjects when each subject walked 400 to 500 strides at preferred speed for 9 minutes [1]. These values of *α* and *β* showed that the time series of stride intervals in human walking exhibits long-range correlations. Fig. 4 shows *α* and *β* obtained from representative data of 500 strides of the model walking when *θ*_0_ was *π*/6 and *π*/12 radian. When *θ*_0_ was *π*/6, the two indices, *α* and *β*, were different from those of uncorrelated white noise or the shuffled data, providing a slight indication of a long-range correlation. Evidence of a long-range correlation became more prominent when *θ*_0_ was reduced to *π*/12; the scaling exponents *α* and *β* were noticeably different from those of uncorrelated white noise or the shuffled data and approached those of human walking. As seen in the bottom panels of Fig. 4 B, the structure of the stride interval time series changed due to shuffling. Fig. 5 A shows the distribution of *α* and *β* for 20 simulations of 500 strides in each of four different cases –infinitesimal *θ*_0_, *θ*_0_ = *π*/12, *θ*_0_ = *π*/6, and normally distributed randomized stride intervals. As *θ*_0_ approached zero and the walking model became a rolling disk, the time series of stride intervals approached Brownian noise. With *θ*_0_ = *π*/12, the distributions of *α* and *β* reproduced those of normal human walking with long range correlations, whereas the indication of long-range correlations became less evident as *θ*_0_ increased to *π*/6. Fig. 5 B compares the distributions *α* and *β* for *θ*_0_ = *π*/12 and *θ*_0_ = *π*/6 with those of the shuffled data. In both cases of *θ*_0_ = *π*/12 and *θ*_0_ = *π*/6, the scaling exponents were significantly different from those of the shuffled data which were indistinguishable from *α* and *β* of uncorrelated white noise. This confirms that sequential ordering gave rise to the long-range correlation; a property of the stride interval distribution is not the origin of long-range correlations for this model.

**Figure 6:**
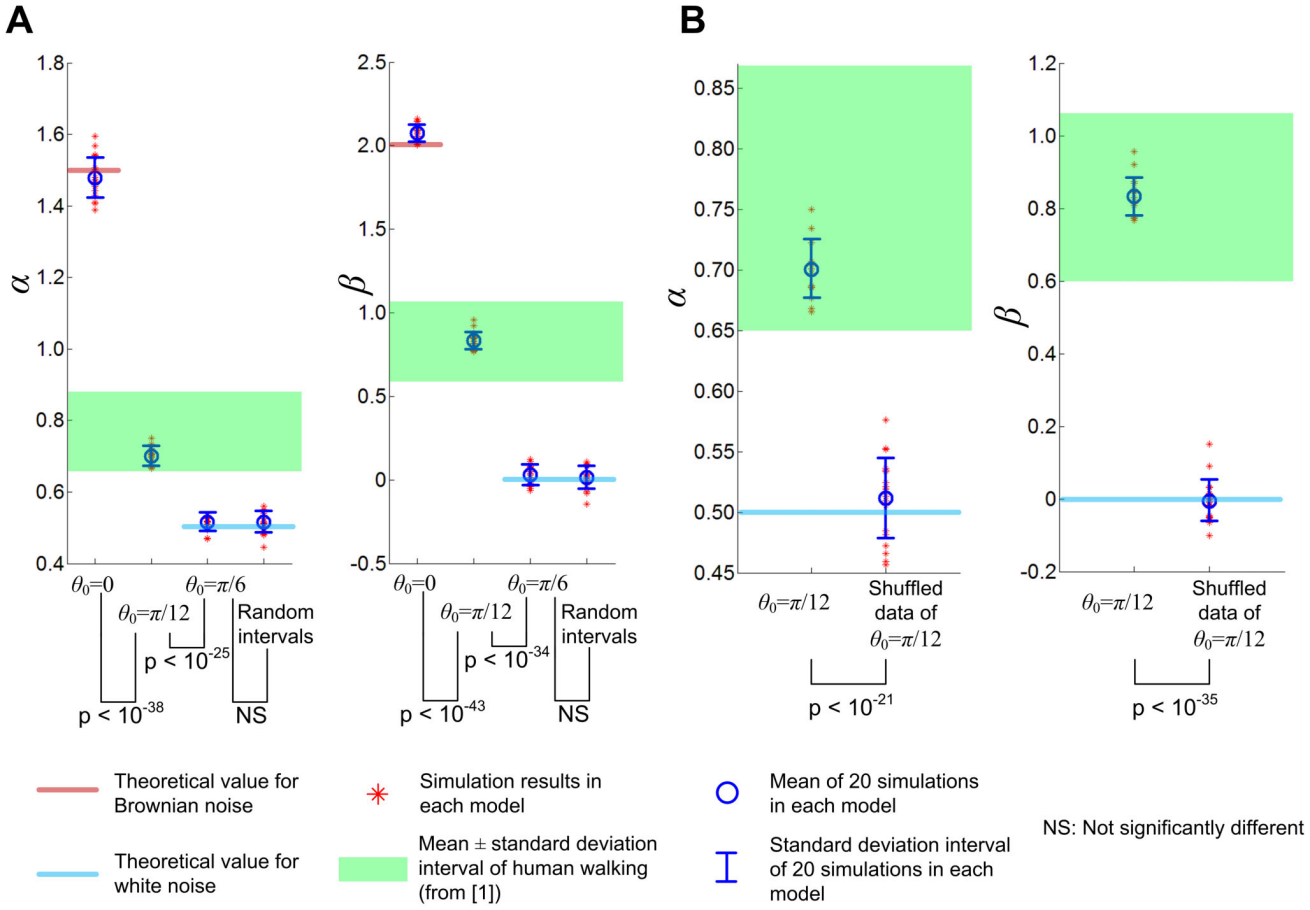
Distribution of scaling exponents *α* and *β* when the model walked 3,000 strides. In A, the scaling exponents *α* and *β* were evaluated for each of 20 simulations. When *θ*_0_ = *π*/6, *α* and *β* are no longer different from those of uncorrelated noise; the large stride number has attenuated the long-range correlation. In contrast, when *θ*_0_ = *π*/12, the stride intervals still present evident long-range correlations with similar *α* and *β* to those observed in human walking. When *θ*_0_ approaches zero and the model has marginal orbital stability, the scaling exponents remain close to those of Brownian noise regardless of the large stride number. In B, the distributions of *α* and *β* for *θ*_0_ = *π*/12 are compared with those of the shuffled time series. (Note the change of plot scale.) As in 500 stride walking, the scaling exponents of the original time series are significantly different from those of the shuffled data which are statistically indistinguishable from *α* and *β* of white noise.

**Figure 7:**
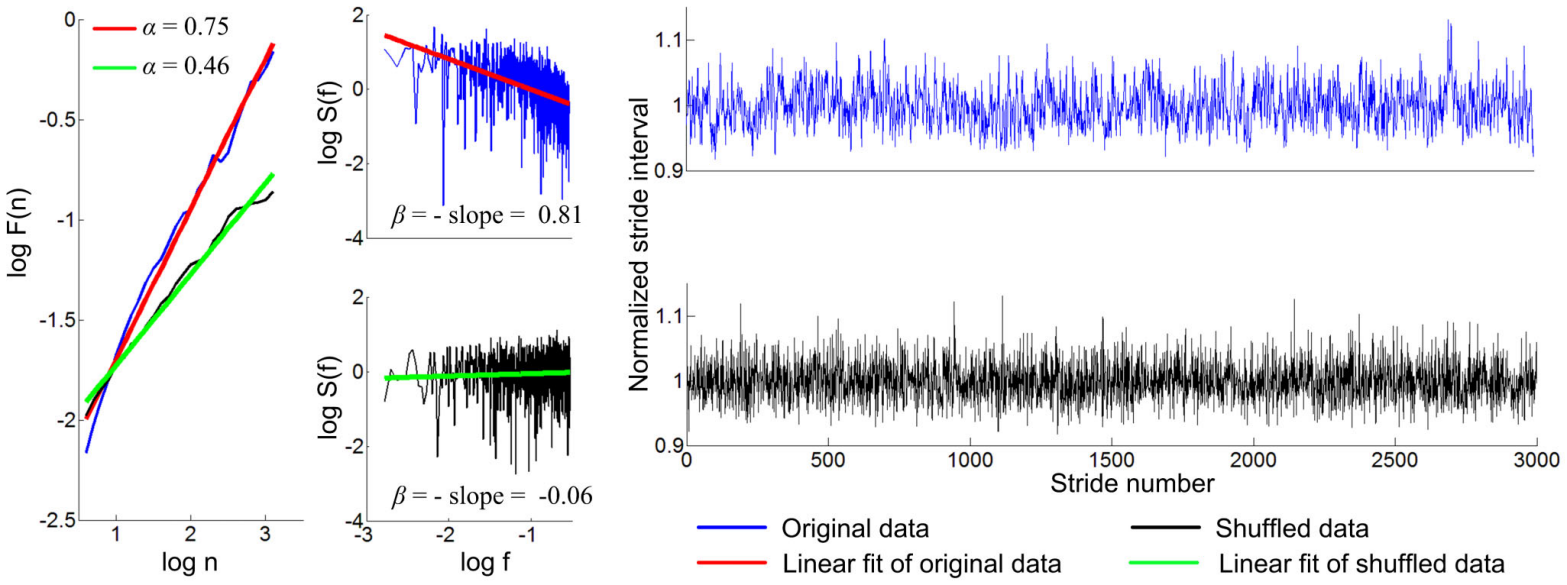
Scaling exponents *α* and *β* for 3,000 strides walking with *θ*_0_ = *π*/12. Exponents *α* and *β* are still similar to those of human walking with long-range correlations, and clearly different from those of the shuffled data. Also in the right panels, it is visible that shuffling changed the structure of the time series of normalized stride intervals (stride intervals divided by the mean value).

Fig. 6 A shows distribution of *α* and *β* for 20 simulations of 3,000 strides. For the case of *θ*_0_ = *π*/6, the indication of long-range correlations disappeared due to the increased stride number; statistical tests concluded that *α* and *β* were not significantly different from 0.5 and 0, respectively. On the other hand, the model with *θ*_0_ = *π*/12 still showed clear evidence of long-range correlations; *α* and *β* were similar to those of human walking with long-range correlations with significant difference from those of white noise. Fig. 6 B compares the scaling exponents of 3,000 strides with those of the shuffled counterparts when *θ*_0_ = *π*/12. As in the 500 strides walking, statistical analysis confirmed that the persistent long-range correlation was due to sequential ordering rather than a specific distribution of the stride intervals. Fig. 7 shows *α* and *β* of representative data of 3,000 strides when *θ*_0_ was *π*/12. The left panels in Fig. 7 additionally demonstrate that the structure of the time series changed after shuffling.

**Figure 8:**
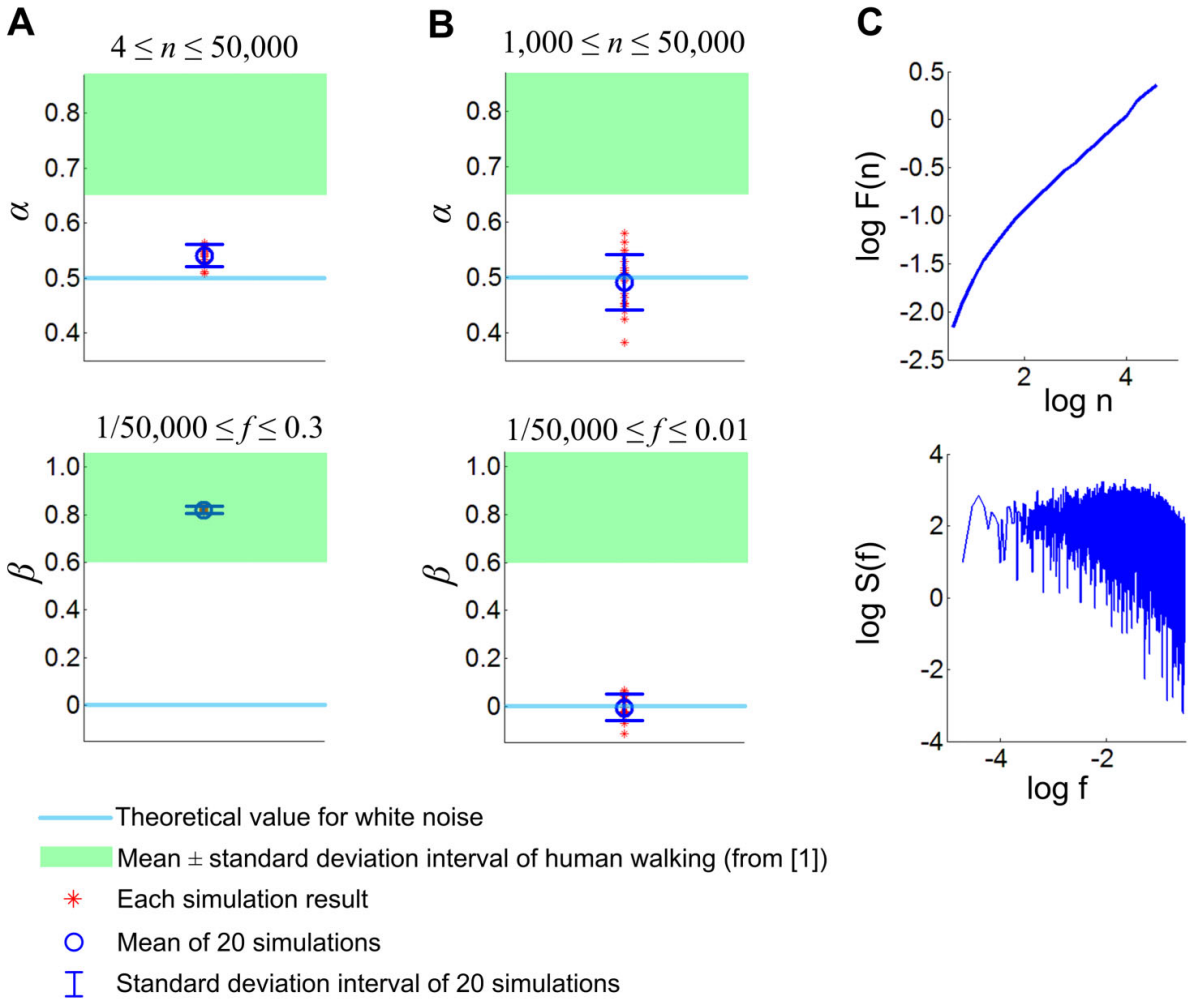
Scaling exponents *α* and *β* for 100,000 stride walking with *θ*_0_ = *π*/12. The scaling exponents are significantly different from those of the uncorrelated noise, but the evidence of long-range correlations is less prominent. In A, *α* is close to that of white noise though *β* is similar to that of human walking with long-range correlations. In B, the exponents are evaluated with window sizes larger than 1,000 strides (1,000≤*n*≤100,000/2, and 2/100,000≤*f*≤0.01); the exponents are not significantly different from those of white noise. C shows the representative data. On the whole, the local slope of the curve relating log *F*(*n*) and log *n* decreases as window size *n* increases.

Fig. 8 shows the simulation results of even longer walking series: 20 realizations of 100,000 stride walking with *θ*_0_ = *π*/12. Statistical analysis concluded that the scaling exponents were still significantly different from those of uncorrelated noise, but the evidence of long-range correlations became less prominent (Fig. 8 A); though *β* was similar to that of human walking *α* became much closer to that of white noise. When we evaluated *α* and *β* with window sizes larger than 1,000 strides (1,000≤*n*≤100,000/2, and 2/100,000≤*f*≤0.01), the exponents approached those of uncorrelated white noise (Fig. 8 B). Fig. 8 C shows representative data of 100,000 strides. Overall, the curve relating log *F*(*n*) and log *n* appears to be slightly convex upward, which explains the decrease of *α* with large window size *n*.

To summarize, the time series of stride intervals of the model showed long-range correlations up to thousands of strides when its orbital stability was moderate though the evidence of long-range correlations became less noticeable when the stride number became much larger up to 100,000. On the other hand, the time series of stride intervals approached uncorrelated random process much more quickly when the increased *θ*_0_ resulted in faster convergence to the nominal limit cycle, making the memory of the dynamics shorter.

## Discussion

The long-range correlations observed in the stride intervals of human walking have received attention because they may quantify locomotor deficits as well as healthy human walking [3], [4], [5]. Though various models have been developed to account for the origin of these long-range correlations, their essential underlying neuro-mechanical origin has not been established. Generally, long-range correlation implies fractal-like behavior that may emerge from chaos, and nonlinear oscillators like CPGs can result in chaos. This explains why most of the previous models involved nonlinear neural oscillators or nonlinear biomechanics that can generate chaos to reproduce the long-range correlations [1],[11],[12],[13],[14]. Here, in contrast to the previous studies, we reproduced the long-range correlations in a walking model that was deliberately simplified so that it could not exhibit chaos. We believe it provides a plausible explanation of the physical origin of long-range correlations.

To reproduce the long-range correlations, we added stochastic noise to a simple walking model described in [16]. As proven in Appendix S1, the model cannot exhibit chaotic behavior. This is an important distinction from the model by Garcia et al. [15]. Gates et al. added stochastic noise to the model presented by Garcia et al. [15] and showed that the model could exhibit long-range correlations, suggesting that nonlinear biomechanics may contribute to the long-range correlations [14]. In fact, Garcia et al. already showed that their original model could exhibit chaotic and fractal behaviors [15], which may result in long-range correlations. In contrast, the work presented here clearly shows that even a highly simplified peripheral mechanism that cannot produce any chaotic or fractal behavior may exhibit long-range correlations when combined with stochastic noise.

Noise exists in various levels of biological systems [25], [26], [27], and has been used to explain experimental observations in some human motor tasks [28], [29]. In fact, it has also been suggested that specific structures of noise may provide explanations of long-range correlations. For example, autoregressive fractionally integrated moving average (ARFIMA) models and Markov models with specific distributions may reveal long-range correlations [30], [31]. However, any physiological origin of those specific distributions of variability in human rhythmic movements has not been proposed. Here, we suggest that a physical mechanism that is fundamental in general rhythmic movements may give rise to the long-range correlation when combined with the ubiquitous noise. Orbital stability is essential; practically, we cannot maintain or observe any unstable periodic motion. Therefore, any model of rhythmic movements that cannot exhibit orbital stability should be regarded as over-simplified in this context. In fact, human walking has moderate orbital stability; we recover our preferred walking motion after any small and momentary perturbation, but we do not recover immediately. Previous experimental studies quantitatively support this [19], [32]. We showed that noise combined with this moderate orbital stability can reproduce the observed long-range correlation up to thousands of strides without any further mechanism like a CPG, chaos or an exotic distribution of the noise. The two minimal components – common noise and essential orbital stability – may be sufficient.

The long-range correlations may be interpreted as evidence that there exists a “memory” in the dynamic process of walking so that the current stride can affect a future stride even after many strides [1]. Based on this, we proposed a hypothesis that long-range correlations in stride intervals are related to the orbital stability of a limit cycle. A strongly stable limit cycle, by rapidly attracting a perturbed system to the nominal limit cycle, allows the perturbation to be forgotten quickly. In contrast, a weakly stable limit cycle allows an effect of perturbation to persist for a long time, resulting in long-lasting memory or long-range correlations. In a limiting case where the system has marginal stability, a random perturbation at the current stride will persist forever. If successive perturbations are uncorrelated, the stride intervals approach Brownian noise, the time-integration of white noise. Our model validated these proposals. Long-range correlations were evident up to thousands of strides when the model had relatively weak orbital stability, whereas the stride intervals approached uncorrelated white noise more quickly when the stability was relatively strong. In the limit as the model became a rolling disk with marginal stability, the history of cycle durations approached Brownian noise.

**Figure 9:**
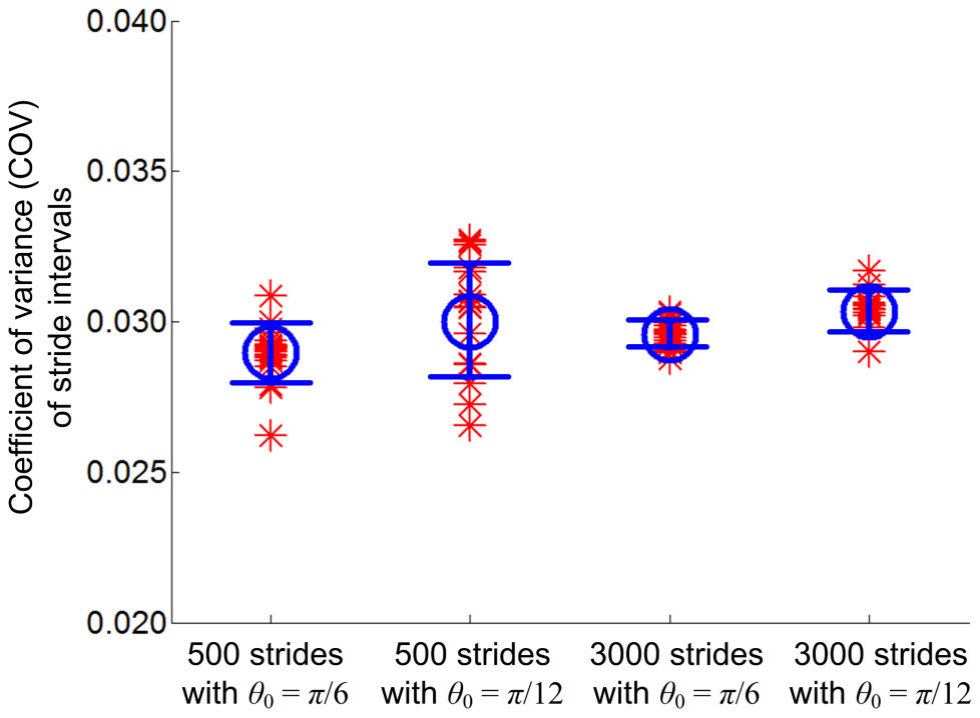
Distribution of coefficient of variance (COV). In both cases of *θ*_0_ = *π*/6 and *θ*_0_ = *π*/12, the distribution of COV obtained from each set of 500 or 3,000 strides is not significantly different from 3%, the COV of stride intervals observed in normal human walking. The circle and error bar indicate the mean and the standard deviation interval, respectively.

The indices of long-range correlations of the model quantitatively match those of human walking obtained in [1]. When *θ*_0_ = *π*/12, *α* and *β* approach those of human walking (Fig. 4 and 5). In normal human walking, the hip flexion angle with respect to a vertical line at the leading foot contact (represented by *θ*_0_ in the model) is close to 15 degrees or *π*/12 [33]. The noise level of each model was also carefully chosen to reproduce the variance of stride intervals observed in human walking. Fig. 9 validates this. In both cases of *θ*_0_ = *π*/12 and *θ*_0_ = *π*/6, the distribution of COV obtained from each simulation was not significantly different from 3%, the COV of stride intervals in normal human walking. This is another contrast between our model and the model by Gates et al. [14]. Due to the limited size of its basin of attraction, the variability exhibited by the model in [14] was much less than that observed in human walking.

In this model, the memory causing the long-range correlation is directly related to mechanics, the linear and angular momentum principles. The original model in [16] conserved angular momentum with respect to the leading ankle at the moment of foot-ground collision. This reduced the speed of the model by a factor of cos2*θ*_0_, and therefore reduced the kinetic energy by cos^2^2*θ*_0_, resulting in a Floquet multiplier of cos^2^2*θ*_0_. When *θ*_0_ = *π*/6 and *π*/12, the Floquet multiplier became 0.25 and 0.75 respectively. When *θ*_0_ = *π*/6, 10 successive steps reduced a perturbation with a magnitude of unity to (0.25)^10^<10^−6^, meaning that the perturbation was rapidly forgotten due to the “short memory” of the system. When *θ*_0_ = *π*/12, 10 successive steps reduced the same perturbation to (0.75)^10^ = 0.056, meaning that more than 5% of the perturbation effect persisted after 10 successive steps.

In the limit as *θ*_0_ approaches zero, the model approaches a rolling disk with mass *m* and zero moment of inertia. In this case, the stability becomes marginal, and any effect of a perturbation can never be forgotten. The current stride interval is determined by the initial condition and the accumulated effects of the perturbations up to the current stride. Therefore, if a perturbation of uncorrelated white noise is added per stride, the time series of stride intervals is expected to approach Brownian noise. Details are in Appendix S1.

The other limiting case is when the model has no “memory” or zero Floquet multiplier. If the attraction to the nominal limit cycle is so strong that no previous stride can affect the current one, the stride interval should be determined purely by the actuation of the current stride. Therefore, uncorrelated variability in ankle actuation will result in uncorrelated variability in stride intervals. As shown in Fig. 6, *θ*_0_ of *π*/6 already brings the stride intervals close to uncorrelated noise in 3,000 strides; *α* and *β* are not significantly different from those of randomized stride intervals or white noise.

Assuming that the dynamics of human locomotion is between these two extremes – a limit cycle with marginal stability and a limit cycle with zero Floquet multiplier – the long-range correlations in stride intervals become understandable rather than counter-intuitive. If the maximum Floquet multiplier has a magnitude of unity and every stochastic fluctuation affects future strides, indices *α* and *β* approach 1.5 and 2 respectively. If the Floquet multiplier is close to zero and the current stride is affected only by the current fluctuation, indices *α* and *β* approach 0.5 and 0 respectively. In fact, the indices *α* and *β* are between these two extremes in human walking [1]. Experimental studies reported that the maximum Floquet multiplier of human walking was also between the two extremes of 0 and 1 [19], [32]. Dingwell and Kang reported that the average magnitude of the maximum Floquet multiplier was between 0.7 and 0.8 for over-ground walking when the multipliers were measured using hip, knee or ankle angles [19]. This value is comparable to the Floquet multiplier of our model with *θ*_0_ of *π*/12. Hurmuzlu and Basdogan originally measured Floquet multipliers of human walking in 18 generalized coordinates; they reported that all the 18 Floquet multipliers were inside a unit circle on the complex plane, and the average magnitudes were between 0.337 and 0.395 [32]. This implies that the magnitude of the maximum Floquet multiplier is less than unity but larger than the reported average, which is consistent with moderate orbital stability.

It is noteworthy that the long-range correlation that our model reproduces is different from the long-range correlation due to self-similarity in infinite time series. Scale-free self-similarity that frequently arises from chaos induces constant scaling exponents regardless of the cycle number, whereas the long-range correlation due to asymptotic orbital stability cannot persist for infinite stride numbers. As shown in Fig. 8, the memory due to asymptotic orbital stability cannot remain effective after a sufficiently large number of strides. This leads to an open question whether the long-range correlation of human walking may also be diluted after a large number of strides. Unfortunately, it would be challenging to test experimentally whether human stride intervals show constant scaling exponents up to 100,000 strides that approximately correspond to 30 hours of walking. In this study, we show that uncorrelated stochastic noise combined with human-like orbital stability can induce scaling exponents similar to those observed in human walking up to thousands of strides. It seems highly plausible that the observed orbital stability of human walking contributes to the observed values of scaling exponents in our routine walking of hundreds or thousands of strides.

The model used in this study neglected numerous aspects of human walking to maximize simplicity. Physiological and anatomical realism was ignored by assuming a point mass and massless legs. Complicated stabilizing mechanisms were simplified to minimal afferent feedback regulating leg angle and state-determined ankle actuation, making the orbital stability a function of leg angle alone. In real human locomotion, various feedback mechanisms with or without supra-spinal control may contribute to stability. For example, human walking seems to require active control to stabilize lateral motion, a factor that was deliberately ignored in this study [34]. In addition, differences in orbital stability between over-ground and treadmill walking suggest that human walking stability may not be purely determined by the mechanics of the periphery [19]. The several sources of variability in human movement were simplified to variability of a single parameter, *k*. Variability in human movement originates from central nervous systems like the basal ganglia and premotor cortex as well as from recruitment of motor units [25], [26], [27]. In this study stochastic noise was added only to the ankle actuation constant. At best it reflects the ubiquitous noise of the motor system but other noise sources may be equally important.

Despite these limitations, this study identifies a physical interpretation of the origin of long-range correlations in the stride intervals of human walking. Stochastic noise is ubiquitous in the central nervous system and the peripheral sensory-motor systems participating in human movement [25], [26], [27]. When this stochastic noise is “filtered” through a system that exhibits a finite orbital stability – whether the stability results from mechanics of the periphery, neural control or a combination of both – the stochastic noise will be partly forgotten as stride number increases. This imperfect but nonzero memory may contribute to the long-range correlations observed in stride intervals of human walking. Whether and how the orbital stability actually affects the long-range correlations in stride intervals of human walking would be an interesting topic for further experimental research.

Long-range correlation is commonly regarded as a signature of fractal-like behavior, and chaotic behavior is frequently observed in nonlinear oscillators. Due to the frequent concurrence of chaos and fractals, the long-range correlations in stride intervals have been considered as evidence that nonlinear oscillators like CPGs play a prominent role in human walking. Consequently, most of the previous models have assumed a dominant role of CPGs in human walking; CPGs determine the stride intervals, and the motor system exactly executes the command [1], [11], [12], [13]. A crucial deficiency in this argument is that there is no direct evidence that a CPG plays a prominent role in upright human walking. The locomotor-like movements evoked by spinal stimuli have been observed only in a gravity-neutral position, rendering it difficult to generalize the results to normal human walking [35], [36]. On the contrary, some experimental evidence suggests that a CPG in human spinal circuitry has been largely suppressed in adult human locomotor control [37], [38]. To reiterate, experimental studies reported fractal-like variability in human walking; modeling studies proposed specific CPGs as the entire source of the specific structure of the variability; but there is no experimental evidence that supports the assumption of the models.

Our results address this important deficiency. First, we showed that even a non-chaotic walking model without a CPG may yield long-range correlations in stride intervals. Second, the limit cycle had to be weakly attracting for the model to reproduce the long-range correlations observed in human walking. This suggests that any CPG that may underlie human walking may be weakly attracting or weakly coupled to other locomotor systems. Contrary to common hypotheses, long-range correlations in stride intervals may not imply a strongly attracting rhythmic primitive such as a neural CPG; a weak attractor that allows long memory may give rise to the long-range correlations in human stride intervals.

## Supporting Information

Appendix S1 **This appendix presents mathematical proofs of the following two: 1) the model has a unique and globally attracting periodic gait, and therefore, it cannot exhibit a chaotic behavior; 2) the time series of the cycle durations of the model approaches Brownian noise with infinitesimal** ***θ*** **_0_.**
(DOCX)

Figure S1 **A graphical illustration of monotonic convergence to the fixed point.** The intersection of *y* = *x* and *y* = *g*(*x*) corresponds to the period-one gait or the fixed point, *x_fixed_*. Any initial condition of *x* should converge to *x_fixed_* monotonically following the green arrows. This precludes the model from exhibiting period-*n* (*n*≥2) gaits or chaos.
(TIF)
