# Supplementary material for: Long-Range Correlations in Stride Intervals May Emerge from Non-Chaotic Walking Dynamics
Source: PLoS One. 2013 Sep 23;8(9):e73239. doi: 10.1371/journal.pone.0073239 (PMC3781160; doi:10.1371/journal.pone.0073239)
Supplement: Appendix S1 — This appendix presents mathematical proofs of the following two: 1) the model has a unique and globally attracting periodic gait, and therefore, it cannot exhibit a chaotic behavior; 2) the time series of the cycle durations of the model approaches Brownian noise with infinitesimal θ 0. (DOCX) [file pone.0073239.s001.docx]

### **Appendix S1**

### **Uniqueness and Stability of the Periodic Gait of the Model**

### Here, we prove that the model cannot have period-n (n ≥ 2) gaits in which the model recovers its state after n steps.

Using the work-energy principle, , where is the right after *i*^th^ foot-ground collision. The map whose input *x* is the angular speed of a step and output *y* is the angular speed of the next step can be defined as$y=g(x)=-\frac{\cos2\theta_{0}}{L}\sqrt{(Lx)^{2}+\frac{2W}{m}}$ , where $W=\frac{k}{2}(\theta_{0}+\mu-\frac{\pi}{2})^{2}$ which is the work done by the ankle torque.

The period-one gait is the fixed point of the map *g*(*x*), and should satisfy *x* = *g*(*x*). There is a unique positive solution of this equation, which is

$x_{fixed}=\frac{\cos2\theta_{0}}{L}\sqrt{\frac{2W}{m(1-\cos^{2}2\theta_{0})}}$ . (S1)

The derivative of the map becomes

$\frac{dy}{dx}=\cos2\theta_{0}\frac{x}{\sqrt{x^{2}+\frac{2W}{mL^{2}}}}$ . (S2)

Therefore,

$\left.\frac{dy}{dx}\right|_{x=0}=0$, and $\left.\frac{dy}{dx}\right|_{x\to\infty}=\cos2\theta_{0}<1$. (S3)

From (S1) and (S2),

$\left.\frac{dy}{dx}\right|_{fixed}=\cos^{2}2\theta_{0}$. (S4)

This corresponds to the Floquet multiplier. If *θ*_0_ *≥ π*/4, no momentum in the current step can be transferred to the next step after foot-ground collision. Moreover, *θ*_0_ *≥ π*/4 means that the angle between the two legs (2*θ*_0_) is larger than 90 degrees, which is far beyond realistic human walking. Therefore, we confine *θ*_0_ between 0 and *π*/4. The second derivative of the map becomes

$\frac{d^{2}y}{dx^{2}}=\cos2\theta_{0}\frac{\frac{2W}{mL^{2}}}{(x^{2}+\frac{2W}{mL^{2}})^{\frac{3}{2}}}>0$. (S5)

From (S2) ~ (S5), $\frac{dy}{dx}$ is monotonically increasing from 0, becomes cos^2^2*θ*_0_ at the fixed point, and asymptotically approaches cos2*θ*_0_ as *x* increases. For all positive initial values of *x*, and for all *θ*_0_ between 0 and *π*/4, $\frac{dy}{dx}$ is between 0 and 1. This guarantees that any initial condition of *x* should converge to *x_fixed_* *monotonically*. A graphical illustration is shown in Fig S1.

If the model has period-*n* (*n* ≥ 2) gaits, any set of *n*+1successive steps should exhibit a non-monotonic sequence of *x* in those gaits. However, a non-monotonic sequence is not compatible with this map as shown above. Therefore, the model cannot have period-*n* (*n* ≥ 2) gaits. Neither period-doubling nor chaos is possible for this model.

**Stride Intervals Approach Brownian Noise with Infinitesimal *θ*_0_**

As in Fig 3, let *v_i_*, *T_i_*, *L* and *F* be the velocity of the disk at the end of *i*^th^ cycle, duration of *i*^th^ cycle, radius of the disk, and the stochastic force applied to the center of mass while the disk was rolling from 0 to *π* radian respectively. The energy at the (*i*+1)^th^ cycle equals work done by the perturbation force *F* plus the energy at *i*^th^ cycle;

$\frac{1}{2}mv_{i+1}^{2}=\pi LF+\frac{1}{2}mv_{i}^{2}$ , or $v{}_{i+1}=\left(\frac{2\pi LF}{m}+v_{i}^{2}\right)^{\frac{1}{2}}$ . (S6)

Because *F* is small enough to keep the COV of 500 successive stride intervals at 3%, using Taylor expansion around *v_i_*,

$v{}_{i+1}=v_{i}+\frac{\pi L}{mv_{i}}F+O(F^{2})$, or $v{}_{i+1}\cong v_{i}+\frac{\pi L}{mv_{i}}F$. (S7)

The duration of a cycle is

$T{}_{i+1}=\text{ duration from 0 to }\pi+\text{duration from }\pi\text{ to 2}\pi=\frac{2\pi L}{v_{i}+v_{i+1}}+\frac{\pi L}{v_{i+1}}$. (S8)

Because the variance of *v_i_* is small enough to keep the COV of *T_i_* at 3% up to 500 cycles, combining (S7) and (S8),

$T{}_{i+1}\text{ }\cong\frac{2\pi L}{v_{i+1}}\cong\frac{2\pi L}{v_{i}+\frac{\pi L}{mv_{i}}F}$ .

Assuming small *F* and using a Taylor expansion,

$T{}_{i+1}\text{ }\cong\frac{2\pi L}{v_{i}}-\frac{2\pi^{2}L^{2}}{mv_{i}^{3}}F\cong\text{ }T{}_{i}-\frac{2\pi^{2}L^{2}}{mv_{i}^{3}}F$.

Considering the small variance of *v_i_*,

$T{}_{i+1}\text{ }-\text{ }T{}_{i}\text{ }\cong\text{ constant}\times F$,

where *F* is a random variable from a normal distribution with zero mean. This shows that the time series of the cycle durations approaches Brownian noise.
