# Supplementary figures and images for: Long-Range Correlations in Stride Intervals May Emerge from Non-Chaotic Walking Dynamics
Source: PLoS One. 2013 Sep 23;8(9):e73239. doi: 10.1371/journal.pone.0073239 (PMC3781160; doi:10.1371/journal.pone.0073239)

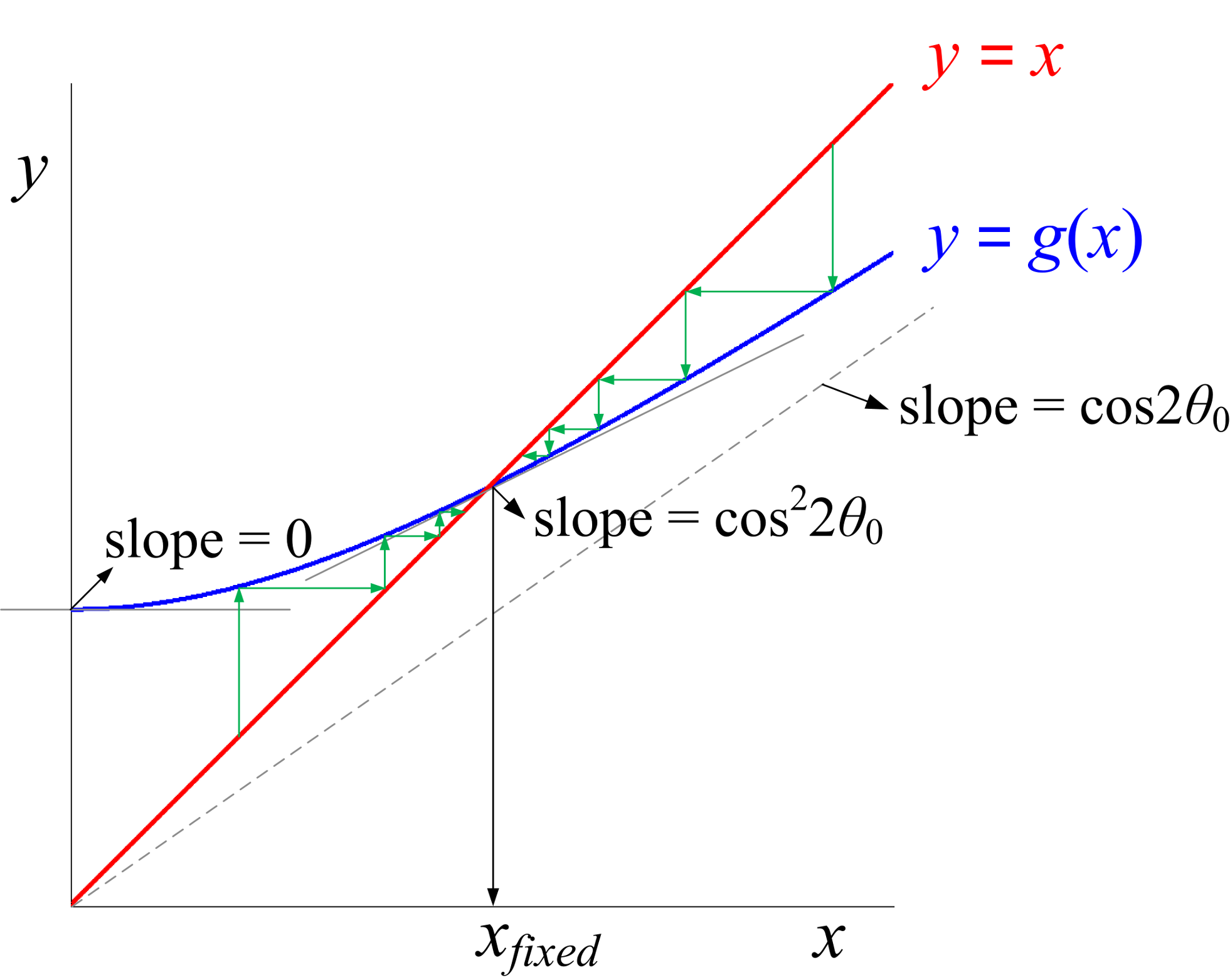

Supplement: Figure S1 — A graphical illustration of monotonic convergence to the fixed point. The intersection of y = x and y = g(x) corresponds to the period-one gait or the fixed point, xfixed. Any initial condition of x should converge to xfixed monotonically following the green arrows. This precludes the model from exhibiting period-n (n≥2) gaits or chaos. (TIF) [file pone.0073239.s002.tif]
